# Diagnosis and Management of Lymphatic Disorders in Congenital Heart Disease

**DOI:** 10.1007/s11886-020-01405-y

**Published:** 2020-10-10

**Authors:** Benjamin Kelly, Sheyanth Mohanakumar, Vibeke Elisabeth Hjortdal

**Affiliations:** 1 Department of Cardiothoracic and Vascular Surgery, Aarhus University Hospital, Aarhus, Denmark; 2 Department of Clinical Medicine, Aarhus University, Aarhus, Denmark; 3 Department of Cardiothoracic Surgery, Rigshospitalet, Copenhagen, Denmark

**Keywords:** Lymphatic complications, Congenital heart disease, Chylothorax, Prolonged effusion, Protein-losing enteropathy, Plastic bronchitis

## Abstract

**Purpose of Review:**

Lymphatic disorders have received an increasing amount of attention over the last decade. Sparked primarily by improved imaging modalities and the dawn of lymphatic interventions, understanding, diagnostics, and treatment of lymphatic complications have undergone considerable improvements. Thus, the current review aims to summarize understanding, diagnostics, and treatment of lymphatic complications in individuals with congenital heart disease.

**Recent Findings:**

The altered hemodynamics of individuals with congenital heart disease has been found to profoundly affect morphology and function of the lymphatic system, rendering this population especially prone to the development of lymphatic complications such as chylous and serous effusions, protein-losing enteropathy and plastic bronchitis.

**Summary:**

Although improved, a full understanding of the pathophysiology and targeted treatment for lymphatic complications is still wanting. Future research into pharmacological improvement of lymphatic function and continued implementation of lymphatic imaging and interventions may improve knowledge, treatment options, and outcome for affected individuals.

## Introduction

**Fig. 1:**
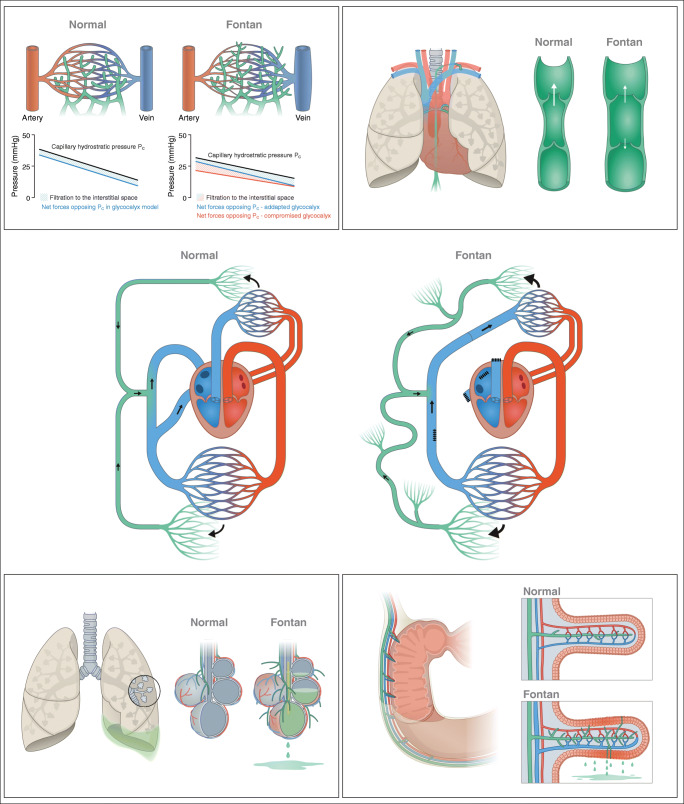
Center: overview of the systemic and pulmonary circulation and the lymphatic system. Lymphatic flow is unidirectional from the capillary bed to the subclavian vein and driven by contractions of the lymphatic vessels. In the Fontan circulation, the subpulmonary ventricle is bypassed, increasing central venous pressure and thoracic duct afterload. The thoracic duct is dilated and tortuous with multiple collaterals. Left top: Capillary filtration in a normal circulation and a Fontan circulation. Under steady state, the revised Starling dictates extravascular filtration throughout the capillary bed. The hemodynamic changes of the Fontan circulation increase filtration (area between lines). The filtration-regulating function of the glycocalyx may adapt to hydrostatic changes and minimize filtration or be compromised resulting in increased filtration. Left bottom: the anatomical course of the thoracic duct. Increased central venous pressure may compromise emptying back into the blood circulation and cause changes to the lymphatic vessels. Normal: a collecting lymphatic vessel with valves securing unidirectional flow during contractions. Fontan: a lymphatic vessel with dilated lymphangions reducing the efficiency of contractions and causing insufficient valves. Right top: duodenum and draining lymphatic vessels. Normal: duodenal villi with blood vessels and lymphatic vessels. Fontan: edematous and inflamed intestinal wall. Multiple dilated lymphatic collaterals. Lymphatic vessels may perforate into lumen leaking lymphatic fluid into the intestines causing protein-losing enteropathy. Right bottom: airways and lungs including draining lymphatic vessels. Normal: small airways and alveoli with blood and lymphatic vessels. Fontan: increased extravascular filtration. Dilated and multiple lymphatic collaterals. Leakage below to pleural cavity and leakage into airways resulting in cast production and plastic bronchitis in the case of inflammation. Illustrations courtesy of Ken Kragsfeldt, Aarhus University

The lymphatic vasculature is widely distributed throughout the entire body. In health, it functions as a unidirectional drainage and transport system originating in the interstitial space and terminating with the return of the lymphatic fluid back into the great veins of the neck or thorax. The initial lymphatic capillaries are composed of a single layer of lymphatic endothelial cells with interstitially anchored filaments preventing collapse. For initial uptake and transport, the fluid is dependent on favorable pressure gradients to reach the pre-collecting and collecting lymphatic vessels. From here, an increasing concentration of smooth muscle cells intertwiningly weaved around the lymphatic vessel contract in order to propel the fluid forward [1–3]. The initiation of contractions and maintenance of an adequate frequency is complexly regulated. Similar to the heart and intestines, pacemaker cells have been proposed to secure contractions and continuous movement, with ion channels required for depolarization [3–6]. Additionally, both increased tension in the vessel wall and adrenergic innervation have been shown to increase contraction frequency, enabling an estimated tenfold increase in fluid removal if needed [7–9]. Lymphatic valves divide the collecting lymphatic vessels into functional semi-independent segments, lymphangions, with contractile properties comparable with those of heart ventricles. Daily, as dictated by the Starling principle, an estimated 8 L of fluid is filtered out from the blood circulation and into the interstitial space [10]. Traditionally, venous reabsorption was thought to reduce this volume; however, a revision of the Starling principle proposes filtration at steady state to be largely unidirectional, with removal of interstitial fluid being conducted exclusively by the lymphatic system. Additionally, an endothelial lining composed of glycosaminoglycans and proteoglycans, the glycocalyx is heavily involved in the permeability of the vessels and regulation of the filtration [10] (Fig. 1). Thus, increased filtration and/or insufficient lymphatic removal may result in interstitial accumulation of lymphatic fluid in the form of tissue edema or effusions if the fluid transudate into nearby cavities. Besides fluid and protein homeostasis, the lymphatic system is heavily involved in both immune surveillance and uptake of lipids in the intestines [9]. Thus, the composition of most lymphatic fluid reflects these primary functions, with the concentration of lymphocytes, various proteins, and lipids being high [11].

Due to the structural and functional heterogeneity of congenital heart disease (CHD), incidence and prevalence of lymphatic complications are immensely varying within subtypes of malformations. Being often referred to as the forgotten circulation, the etiology behind the development of lymphatic complications remains poorly understood. Generally, the incidence of effusions and other lymphatic complications tend to increase with increasing venous congestion and more severe congenital heart malformations. Accordingly, individuals with tetralogy of Fallot and univentricular heart disease are excessively overrepresented with regards to lymphatic complications in CHD. Sparked primarily by improved lymphatic imaging modalities and the dawn of the field of lymphatic interventions, understanding, diagnostics, and treatment of lymphatic complications have undergone considerable improvements over the last decade. Thus, the current review aims to summarize understanding, diagnostics, and treatment of lymphatic complications in individuals with CHD.

## Lymphatic Complications

In CHD, hemodynamic changes arising from structural abnormalities or their correction may result in increased hydrostatic pressure in parts of the circulation. In the capillary beds, this causes increased filtration of fluid to the interstitial space increasing the necessity for lymphatic removal. Additionally, the increased central venous pressure (CVP) commonly found in some subgroups impedes the emptying of the thoracic duct, adding additional congestion to the already pressured lymphatic system. Consequently, many individuals with congenital heart defects are especially prone to the development of lymphatic complications (Fig. 1). Thus, effusions or chylothorax may develop following the prompt increase in hydrostatic pressure or following an unintended perforation of the thoracic duct or its tributaries during surgery [11, 12]. Protein-losing enteropathy (PLE) and plastic bronchitis (PB) typically develop postoperatively or gradually over time, with the mechanism thought to be a combination of hemodynamic changes resulting in increased CVP, lymphatic congestion, and a lymphatic morphology prone to leakage and development of complications [13]. Similarly, peripheral edema develops when the extravasation of fluid, dictated by changes in hydrostatic or oncotic pressure or altered permeability of the endothelial glycocalyx, exceeds lymphatic removal [10]. Although complications may present in individuals with less complex congenital heart defects and with increased incidence and prevalence alongside genetic syndromes such as Downs, Noonan, and Turner syndrome [14–16], the main perspective of the following will be individuals with tetralogy of Fallot and univentricular heart defects.

## Lymphatic Effusion and Chylothorax

Prolonged pericardial or pleural effusions and chylothorax and chylopericardium may all, given the origin of the fluid, be considered as lymphatic complications. Prolonged effusions are most commonly observed following surgical repair of either tetralogy of Fallot or univentricular heart disease [17]. Depending on the hydrostatic and oncotic pressure gradient and the permeability of the endothelial glycocalyx, serous fluid is filtered from the capillary bed and out into the interstitial space of both the pleural and lung parenchyma. Under normal conditions, fluid flows from the parietal pleura, through the pleural space, to be absorbed by the lymphatic capillaries in the visceral pleura. Similarly, the lymphatic capillaries of the lung take up fluid from the interstitial space of the lung [18, 19]. Changes altering the hydrostatic pressure in the interstitial compartments may cause fluid to stagnate or transude into and accumulate in the low-pressure environment of the pleural space.

Prolonged effusions are reported in between 7.3 and 13.5% of operated tetralogy of Fallot patients and may be viewed as a disruption of the fluid equilibrium [20, 21]. The mechanism behind is likely multifactorial and a combination of changed physiology and the trauma of the operation. Following surgery, some patients develop low cardiac output syndrome with constrictive right ventricle physiology. Although it has been found to be beneficial in the long term and protective against the consequences of pulmonary regurgitation, the restrictive right ventricle physiology is associated with increased right atrial pressure, which along with increased CVP is a risk factor for prolonged lymphatic effusions in the postoperative period [22–25]. Also, the relief of the right ventricular outflow obstruction during operation instantly increases pulmonary blood flow, capillary hydrostatic pressure, and the filtration gradient towards the interstitium. The increased pressure causes fluid to transudate from the lung interstitium through the parietal pleura and accumulate in the pleural space [17, 19]. Being a temporary disruption, over time, the microcirculation may adapt to the changes and reach a new equilibrium with effusions spontaneously resolving.

Similarly, during the creation of a bidirectional cavopulmonary connection or total cavopulmonary connection, the blood flow and the hydrostatic pressure in the pulmonary capillary beds tend to increase, augmenting filtration and risk of effusions, the incidence following correction being 12.6% and 37%, respectively [26]. In the postoperative intensive care unit, effusions may contribute to respiratory insufficiency and increased dependency on mechanical ventilation [11, 27]. In the Fontan circulation, pulmonary blood flow is exquisitely sensitive to changes in intrathoracic pressure. The absence of negative inspiratory pressure during prolonged ventilation may not only reduce pulmonary blood flow and cardiac output but also add to the congestion of the venous system, increasing volume of effusions and impeding emptying of the thoracic duct [28, 29]. Moreover, the lymphatic vessels in these patients are thought to be morphologically and functionally changed, further increasing the tendency to develop effusions [17, 30, 31]. Overall, when ventilating patients experiencing effusions and lymphatic complications, more conservative ventilator settings should be considered, or preferably early extubation when possible.

Chylothorax and chylopericardium are other postoperative lymphatic complications. They are common following traumatic perforation of the thoracic duct or its tributaries during surgery, with leakage accumulating postoperatively. However, it is also observed more sporadically years after surgery. Here, the proposed mechanism is a combination of increased intralymphatic pressure due to increased uptake and impeded return and structural weakness in the lymphatic vessels. Perforation of the endothelial weakness provides a way of decompression for the congested lymphatic system and reroutes fluid into compartments of lower pressure.

Chylothorax occurs with an incidence of 7.4% and 9–24% following surgical repair of tetralogy of Fallot and “surgical palliation” of single ventricles, respectively [21, 32, 33]. Chylothorax has been associated with poorer medium-term outcome and has been speculated to be a sign of lymphatic intolerance to the changed physiology [32]. In addition to congenital heart defects, individuals with Noonan syndrome, Down syndrome, and Turner syndrome also all have increased risk of postoperative chylothorax, with one proposed explanation being abnormal development of lymphatic collaterals, lymphangiectasia. [16, 34–36]

The clinical manifestations of effusions and chylothorax may progress from asymptomatic to the experience of dyspnea, coughing, and chest discomfort with gradually increasing amounts of accumulating fluid [37, 38]. Persisting loss of chyle may result in several deficiencies related to the protein and lipid-rich content of the fluid, and thus, treatment should bear in mind the continuous loss of calories, electrolytes, and volume [38].

Following symptoms, effusions or chylothorax are normally diagnosed with the finding of fluid during ultrasonography or radiographic examination. Commonly, measurement of triglycerides has been used to categorize the fluid as chyle leaking from the lymphatic system. However, as argued above, also fluid filtered out in the lung and transuded into the pleural space may be considered lymphatic fluid. Thus, the content of triglycerides and ratio of triglycerides or cholesterol in pleural fluid compared with serum should, to a greater extent, be viewed as a way of determining the origin of the lymphatic fluid. Thus, triglycerides above the threshold of 110 mg/dl indicate intestinal origin of the lymphatic fluid, confining the leakage to the thoracic duct or its main tributaries. Similarly, low levels of triglycerides indicate an origin of the lymphatic fluid, before the mixing with the lipid-rich fluid flowing in the central thoracic duct [38–40]. Information as to the state of the lymphatic system and the site of lymphatic leakage may be gathered by lymphangiography or lymphoscintigraphy. [41] More recently, magnetic resonance imaging has been applied to visualize the central lymphatic architecture using heavily T2-weighted images. Images may be conducted with or without intranodal or intradermal injection of an oil-based contrast agent. [42, 43] Video-assisted thoracoscopic surgery may also be utilized for direct visualization of the leakage point in chylothorax [37]. In patients with CHD, search for hemodynamic significant stenosis or thrombosis should also be conducted as part of a simultaneous hemodynamic evaluation [13].

While prolonged effusions may resolve following a new steady state between filtration and lymphatic removal, the volume of accumulated fluid may deem treatment necessary. Treatment of effusions and chylothorax evolves around two primary goals: short-term relief of respiratory symptoms by drainage of fluid and long-term prevention of recurrence by treating the underlying cause.

Thoracocentesis and placement of a chest tube will secure immediate relief of respiratory symptoms and maintain continuous drainage. Nonsurgical conservative options for chylothorax include a no-fat diet in order to reduce production of chyle. Medium-chain triglycerides may be added, as these are absorbed directly into the portal vein, bypassing the lymphatic system [37, 44, 45]. Alternatively, conservative treatment may persist of complete enteric rest combined with total parenteral nutrition [46].

In patients unresponsive to conservative nutritional therapy, pharmacological agents such as somatostatin or the synthetic analog octreotide have been reported efficient in treatment [46, 47]. The pathway of function is speculative, with a reduction in both splanchnic blood flow and gastrointestinal secretion, an antagonistic effect on lymphatic vessel function, and reduced uptake of triglycerides being proposed as possible mechanisms [46, 47]. Other treatment options include nitric oxide, etilenephrine, corticosteroids, propranolol, and high positive-end expiratory pressure ventilation [48–52]. However, evidence supporting the efficacy of these treatments remains sparse. Additionally, in both groups, fluid restriction, diuretics, and/or treatment with pulmonary vasodilators may aid in optimizing hemo and lymphodynamics of the circulation and decrease lymphatic production in addition to decreasing thoracic duct afterload. Non-operative management of chylothorax in children is reported successful in > 80% of cases, including patients with chylothorax following cardiothoracic surgery [37].

Traditionally, surgical treatment of chylous leakage has been reliant on thoracic duct ligation or pleurodesis performed via either video-assisted thoracoscopy or open thoracotomy, with pleuroperitoneal shunting reserved for the most persisting cases [37, 53–55]. Similarly, persistent and substantial effusions may also be treated with pleurodesis or shunting [17]. Ideally, prior to/or during surgery the leakage site is located using lymphatic imaging or injection of dye in the lymphatic system intraoperatively. More recently, both transvenous and percutaneous fluoroscopy-guided embolization of the thoracic duct have been described, with dynamic contrast-enhanced lymphatic imaging securing both precision of the intervention and appropriate patient selection [56–58].

## Protein-Losing Enteropathy

PLE is characterized by an abnormal and occasionally profound intestinal loss of lymphatic fluid and protein. In addition to CHD, PLE may be observed secondarily to several other diseases ranging from congestive heart failure to infection and inflammatory bowel disease. In CHD, incidence of PLE has been reported as affecting from 4 to 15% of individuals following Fontan palliation [59–61].

**Fig. 2:**
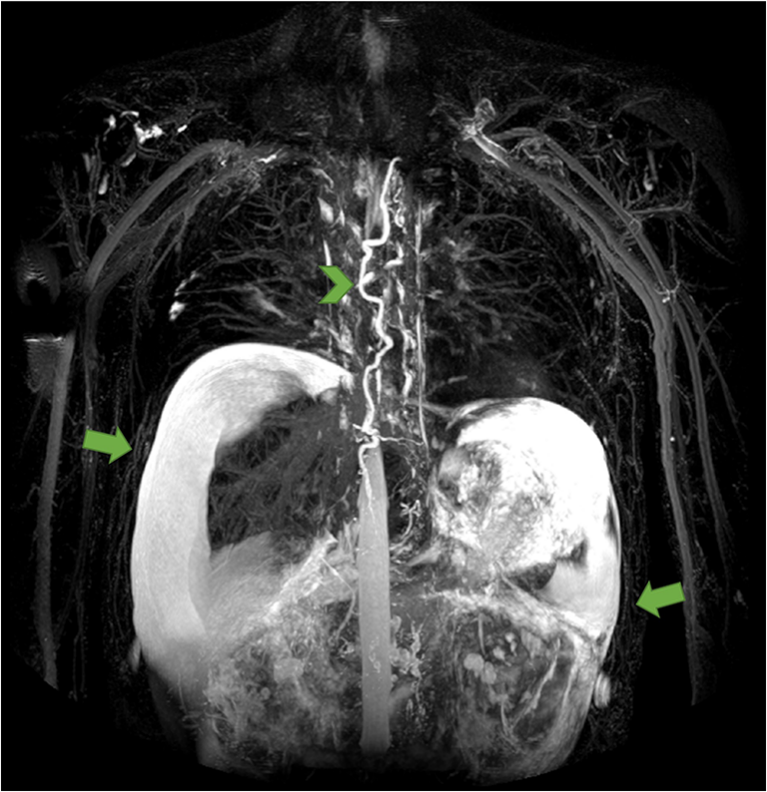
Non-contrast MR lymphography of adult Fontan patient suffering from protein-losing enteropathy. Dilated and tortuous thoracic duct marked by arrowhead. Substantial amounts of abdominal fluid around liver and spleen marked by arrows

The pathophysiology behind the development of PLE is incompletely characterized and with a variety of factors contributing, although the combination and impact of each may vary between cases. Under normal circumstances, the liver and intestines are responsible for the production of 25–50% of the lymphatic fluid flowing in the thoracic duct [62]. In individuals with PLE, lymphangiectasis and dilated lymphatic vessels are common adaptations found in the vessels in the wall of the intestinal tract [31, 63]. An altered lymphatic architecture with vessels adjacent to the intestinal lumen combined with lymphatic congestion and flow obstruction may, in case of increased lymphatic permeability or vessel rupture, result in retrograde lymphatic flow, with protein-rich lymphatic fluid spilling out into the low-pressure environment of the intestines or abdominal cavity (Figs. 1 and 2) [64–66]. Thus, individual variability of the lymphatic architecture may leave some Fontan patients more susceptible to complications and explain why not all congenital heart defects with increased CVP develop lymphatic complications [13].

Other potential mechanisms thought to be involved in the development of PLE in CHD include diminished mesenteric blood flow causing cellular apoptosis and increased permeability of the intestinal wall [67]. Additionally, the low cardiac output state of the Fontan circulation promotes a pro-inflammatory state with increased levels of TNF-α and other inflammatory cytokines which influences the intestinal epithelial barrier [68, 69]. Heparan sulfate and heparan sulfate proteoglycans are both, among other things, involved in the maintenance of endothelial barriers, with heparan sulfate being the most abundant component of the endothelial glycocalyx [70]. The depletion of these glycosaminoglycans from the basolateral surface of cells has been shown to result in fluid and protein leakage [71, 72]. Interestingly, both IFN-γ and TNF-α affect these molecules, and an increase of these inflammatory mediators may induce heparan sulfate loss and a downregulation of heparan sulfate proteoglycan expression tipping the scale and inducing leakage, explaining why the development of PLE often is preceded by a period of infection or inflammation [72, 73].

While PLE often manifests when the protein leakage is substantial and resulting in severe morbidity, it may also exist in a subclinical version. In addition, the loss of protein may be transient with alternating asymptomatic and affected periods. Symptomatic progression of PLE is related to the loss of protein and range from the development of chronic diffuse edema to diminished tissue integrity and impaired wound healing [74]. Impaired synthesis of clotting factors and anti-thrombin may disturb the coagulation cascade, increasing the thromboembolic risk and adding to the already considerable risk of the Fontan circulation. Calcium transport is largely dependent on albumin, and thus, patients with PLE are prone to low bone mineral density secondary to hypocalcemia in addition to impaired growth [74, 75]. In addition to protein and lipids, the lymphatic fluid contains both lymphocytes and immunoglobulins, a persisting loss may result in lymphopenia and impaired immunity [76].

Measurement of an elevated α-1 antitrypsin clearance in a 24-h stool collection is the golden standard for diagnosis of PLE [77]. However, elevation in a single stool sample combined with serum hypoalbuminemia and edema without other identified cause may also be considered diagnostic. Subclinical PLE may be suspected with repeated measurements of slowly declining albumin concentrations. In addition to a general laboratory work-up, both cardiac imaging and cardiac catheterization may be performed for hemodynamic evaluation and a non-contrast MR-lymphangiography to characterize the lymphatic vasculature [13].

The efficacy of pharmacological treatment of PLE is largely case-based, and the lack of randomized controlled trials is universal. Primary treatment aims at decreasing fluid overload through diuretic therapy and preventing hypoproteinemia and low serum oncotic levels through a high-protein, low-fat diet or parenteral nutrition and intravenous albumin [74, 78]. Traditional diuretic therapy may be extended to include an aldosterone receptor inhibitor to further increase natriuresis, limit potassium loss, and potentially play an anti-inflammatory role [79]. Time-limited anti-inflammatory glucocorticoid treatment may be considered as it has been reported to help maintain serum albumin and minimize symptoms of PLE. Due to its high first-pass hepatic metabolism, oral budesonide has been preferred to avoid systemic side effects. However, affected hepatic function is common in the Fontan patients [80] and trouble weaning out of treatment without relapse may still leave treated patients with unwanted short- and long-term side effects [81]. Treatment in the form of either intravenous or subcutaneous unfractionated heparin has been reported to induce remission in PLE patients, with the proposed mechanisms being either an anti-inflammatory effect, stabilization of the cell-matrix and improved glycocalyx barrier function, or through binding and inactivation of IFN-γ and TNF-α alleviating heparan sulfate loss and increasing expression of heparan sulfate proteoglycans in the endothelial wall [82, 83]. PDE-5 inhibitors may be used to reduce pulmonary resistance and CVP, consequently easing lymphatic drainage and potentially increasing mesenteric tissue perfusion [84]. Somatostatin or the analog octreotide may improve symptoms of PLE, a reduction in splanchnic blood flow, gastrointestinal secretion, and antagonistic effect on lymphatic function being proposed as possible mechanisms [85]. Finally, treatment with immunoglobulin to increase oncotic pressure and immune competency [86], loperamide to prevent protein loss in PLE complicated by severe diarrhea [87], intense cardiac rehabilitation and prescribed exercise [88], and dopamine infusion [89] have all been reported to reduce symptoms in small series of patients.

Invasive procedures may secure a more permanent symptomatic relief and should be considered on a case-by-case basis. Late fenestration with the creation of a right to left shunt may increase cardiac output and diminish symptoms at the expense of cyanosis [66, 90]. A lymphatic interventional approach with percutaneous fluoroscopy-guided embolization of liver lymphatic vessel connected to the leaky lymphatic capillaries in the intestinal wall may induce remission in select patients [63]. Similarly to creation of a fenestration, surgical or interventional rerouting of the innominate vein and the thoracic duct inlet to the low-pressure environment of the common atrium may improve lymphatic return and result in symptomatic relief [91–93]. Finally, as a last resort, heart transplantation has been demonstrated to provide consistent relief of PLE with mortality comparable to that of non-PLE Fontan patients, however, due to an often prolonged catabolic course of the complication, some individuals may be found too frail for transplantation [94].

## Plastic Bronchitis

PB is characterized by the formation of thick rubbery casts in the lumen of the airways. Not being exclusively associated with structural CHD, PB may also be observed alongside everything from asthma to various lymphatic diseases, sickle cell disease, and cystic fibrosis. Although rare, and only reported in < 5% of Fontan patients, PB causes severe morbidity in the form of chronic coughing, hypoxemia, expectoration of casts, and asphyxiation [74].

The underlying pathophysiology behind PB in CHD patients is not fully understood. However, recent findings attribute a central role to a pressured and morphologically changed lymphatic system ultimately resulting in lymphatic leakage into the airways [95–98]. Similarly to PLE, the lymphatic system in patients with PB may be thought to be challenged on multiple fronts. The elevated CVP impedes lymphatic return and causes lymphatic congestion. Additionally, the changed lymphatic architecture with the existence of multiple abnormal collaterals and larger more dilated lymphatic vessels may increase risk of leakage, with protein-rich fluid leaking into the low-pressure environment of the airways [30, 31, 98, 99]. Following leakage, as the final step, an inflammatory reaction, preceded by concurrent respiratory infections or individual abnormalities in the inflammatory response causes the fibrin in the lymphatic fluid to cross-link, producing the solid casts pathognomonic of PB (Fig. 1).

Chronic coughing, wheezing, dyspnea, pleuritic chest pain, fever, or respiratory symptoms unresponsive to bronchodilators may lead the clinician to consider a possible PB diagnosis [100–102]. Chest radiographic findings may include signs of concurrent infection, chylous effusion, and/or signs of partial or full atelectasis resulting from airway obstruction [101, 102]. The final diagnosis is confirmed by the production of a bronchial cast either by expectoration or bronchoscopic removal. As previously mentioned, a detailed cardiac work-up containing functional imaging and invasive hemodynamic measurements may aid in providing a more nuanced overview of circulatory challenges [13, 103].

The overall treatment of PB should have two primary goals: an initial improvement of respiratory symptoms and hemodynamics and secondary targeting of venous and lymphatic congestion. Because of the rarity and heterogeneity of the condition, the evidence behind treatment for plastic bronchitis is scarce. Removal of any obstruction under diagnostic bronchoscopy may initially relieve respiratory symptoms [103]. Optimization of hemodynamics and reduction of pulmonary resistance by treatment with pulmonary vasodilators, PDE-5 inhibitors, and potentially combined with endothelin-1 inhibition [103, 104]. Carvedilol may be used to lower end-diastolic blood pressure and increase ventricular filling, and diuretics may be used to reduce excess fluid and venous congestion in the optimization of hemodynamics [74, 103]. Mobilization of casts through treatment with bronchodilators and intensive chest physiotherapy may prove effective [13, 104]. Mucolytic treatment in the form of inhaled N-acetylcysteine or dornase-α may aid in this clearing of airways [104, 105]. By increasing the osmotic gradient and favoring increased dilution of the bronchial mucus, inhaled hypertonic saline may also be efficient [102]. Additionally, nebulized and inhaled tissue plasminogen activator alteplase, tPA may oppose the fibrin crosslinking and aid in the degradation of already formed casts [101, 106]. Unfractionated heparin has also been shown to alleviate symptoms of PB, possibly through both prevention of conversion of fibrinogen to fibrin through increased antithrombin function, through anti-inflammatory properties and perhaps in rebuilding the integrity of the glycocalyx as described above. A similar anti-inflammatory effect of corticosteroids through suppression of numerous mediators and inhibition of both innate and adaptive immune cell response has also been shown beneficial [104, 107]. In addition, treatment with macrolide antibiotics in chronic PB may aid possibly through increased mucosal clearance and its bacteriostatic and anti-inflammatory properties and treatment with leukotriene receptor antagonist have also been reported applied for their anti-inflammatory effect [102, 104].

In addition to pharmacotherapy, dietary changes in form of a low-fat diet in combination with ligation of the thoracic duct have been shown to relieve symptoms or cause resolution in some patients and may be considered [108]. Until recently, Fontan takedown or heart transplantation was considered the last resort in dealing with patients with PB [103, 109, 110]. Similarly to the treatment of PLE, catheter-based percutaneous fluoroscopy-guided embolization may aid in both visualization of leakage and embolization of the lympho-bronchial communication resulting in durable remission and outcomes with low morbidity and mortality [96].

Despite symptomatic relief on specific symptomatic treatment, the detrimental pathophysiology of the Fontan circulation is largely unaltered. Thus, continued pharmacological treatment with pulmonary vasodilators and diuretics may be deemed necessary, or additional surgical interventions to optimize hemodynamics and lymphodynamics may be considered. Late catheter-based fenestration redirects flow and lowers venous congestion [90]. As in patients with PLE, decompression of the thoracic duct by either surgical or interventional rerouting of the innominate vein to the low-pressure environment of the right atrium may relieve lymphatic congestion and improve symptoms as a last resort in severe failing Fontan patients. As with fenestration, the rerouting comes at the cost of cyanosis [91–93].

## Peripheral Edema

Overall, the cause of interstitial accumulation of fluid or edema can be described as an imbalance between potentially four different factors; the hydrostatic- and the oncotic pressure both intravascularly and in the interstitial space, the permeability of the endothelial glycocalyx, and finally the function of lymphatic vessels in removing fluid and protein. Any disturbance to the equilibrium of these forces resulting in a greater microvascular filtration or an attenuation of the lymphatic removal may cause accumulation of fluid and edema [10, 111]. Edema may be due to systemic causes such as cardiac, hepatic, or renal insufficiency or be due to insufficient lymphatic function, the latter characterized as lymphedema.

Edema in patients with CHD is thought to be primarily revolving around circulatory pressure changes and a decrease in cardiac function. However, individuals with concurrent Turner or Noonans syndrome may have inherent genetic defects impairing normal lymphatic vessel development [112].Accordingly, similar morphological lymphatic changes have been associated with the Fontan circulation [99]. Also, the altered univentricular circulation has been shown, over time, to cause multiorgan dysfunction affecting both renal and hepatic function [113, 114]. Both changes that are capable of increasing the probability of peripheral edema through decreased lymphatic function and decreased intravascular oncotic pressure, respectively.

Whatever the cause, the diagnosis of peripheral edema is predominantly based on patient history and clinical findings. Lymphoscintigraphy, lymphangiography, and NIRF imaging may support the diagnosis and help visualize the abnormal route of the lymphatic flow. In addition, duplex ultrasound may reveal chronic venous insufficiency, a common disposition leading to peripheral edema [115].

Treatment and prevention are cause-dependent; optimizing cardiac function and preventing further renal and hepatic deterioration may be relevant within patients with CHD. Persisting edema may be treated with exercise, decongestive physiotherapy, compression stockings, or pneumatic compression therapy [116, 117].

## Summary and Future Directions

Although the recent decade has seen a sparked interest in research into the function of the lymphatic system and its involvement in various complications, a full understanding of the pathophysiology behind the majority of lymphatic complications is still wanting. Particularly within the field of CHD, research is challenged by the heterogeneous nature of the primary heart defects, and the low numbers treated at each center. Accordingly, targets for the current pharmacological treatments are speculative, based on a limited number of patients, and the lack of randomized placebo-controlled trials is universal.

Future continued implementation of lymphatic interventions and increased experience with pre-operative lymphatic risk stratification may improve patient selection and reduce risk of postoperative development of lymphatic complications [98]. Targeted pharmacological treatment of lymphatic complications is wanting and drugs selectively targeting and increasing inotropic and chronotropic lymphatic function may be hypothesized to alleviate congestion and postpone or prevent the development of lymphatic complications, and their discovery should be pursued (Table 1).

**Table 1:** List of management strategies and potential treatments for lymphatic complications

Condition	Strategy	Objective
All		
Diagnostic evaluation		
	Clinical examination	
	Cardiac imaging (echocardiography, MRI, or CT)	Identify anatomic problems and measure hemodynamics
	Cardiac catheterization	Measure hemodynamics (e.g., central venous pressure)
	Lymphatic imaging (MRI lymphangiography)	Characterize lymphatic vasculature, identify leak
	ECG and Holter monitoring	Identify hemodynamically important arrhythmia
Medical management		
	Diuretics	Reduce overhydration and lymphatic congestion
	Aldosterone	Similar to diuretics, reduce inflammatory treat heart failure
	PDE-5 inhibitor	Pulmonary vasodilation, reduce lymphatic congestion
	Endothelin-1 inhibitor	Pulmonary vasodilation, reduce lymphatic congestion
Serous or chylous effusions		
Diagnostic evaluation		
	X-ray	Evaluate fluid existence and volume
	Ultrasound	Evaluate fluid existence and volume
	Thoracocentesis, biochemistry (triglycerides)	Confirmation of chylous content, origin of lymph fluid
	VATS	Localize lymphatic vessel leakage
	Lymphoscintigraphy	Confirm lymphatic vessel leakage
	MR lymphangiography	Localize leakage, visualize potential lymphatic abnormalities
Medical management		
	Somatostatin/octreotide	Reduce chyle production and effusion
	Nitric oxide	Pulmonary vasodilation, reduce lymphatic congestion
	Etilefrine	TD constriction, reduction of lymph flow
	(Corticosteroids)	Anti-inflammatory effect
Other management		
	No-fat diet or total parenteral nutrition	Reduce production of chylous lymphatic fluid
	(High positive-end expiratory pressure ventilation)	Reduce thoracic lymph flow
Surgical management		
	Thoracocentesis	Symptomatic relief
	Pleurodesis	Obliterate pleural space, minimize leakage
	Surgical ligation of TD (open thoracotomy or VATS)	Prevent chyle flow
	TD embolization (percutaneous or venous)	Embolization of TD, prevent flow of lymph
	Pleuroperitoneal shunt	Rerouting of lymphatic fluid, reduce respiratory symptoms
Protein-losing enteropathy		
Diagnostic evaluation		
	24-h α-1 antitrypsin measurement	Confirm diagnosis
	Serum albumin	Alternative confirmation combined with elevated α-1 antitrypsin
	MR lymphangiography	Visualize lymphatic system and potential abnormalities
Medical management		
	Diuretics (including aldosterone receptor antagonists)	Reduce overhydration, anti-inflammatory effect of aldosterone
	Corticosteroids	Reduce inflammation, increase serum albumin
	Unfractionated heparin	Anti-inflammatory, glycocalyx improvement
	PDE-5 inhibitor	Pulmonary vasodilation, reduce lymphatic congestion
	Somatostatin/octreotide	Reduce splanchnic blood flow, chyle production, and lymphatic function
	(Loperamide)	Reduce intestinal motility, increase absorption of proteins
	(Dopamine)	Improved cardiac function, bridge to transplant
Other management		
	No-fat diet or total parenteral nutrition	Nutrition, reduce production of chyle
	Intravenous albumin	Raise serum oncotic pressure
	Intravenous immunoglobulin	Raise serum oncotic pressure, improve immune competency
	(Cardiac rehabilitation and exercise)	Improved hemodynamics, reduced lymphatic congestion
Surgical management		
	Late fenestration	Reduce lymphatic congestion
	Liver lymphatic embolization	Block origin of lymphatic leakage
	TD decompression (surgical or interventional)	Reduce lymphatic congestion, improve cardiac output
	Fontan take-down	Improve cardiac output, reduce lymphatic congestion
	Heart transplantation	Normalize circulation
Plastic bronchitis		
Diagnostic evaluation		
	Bronchoscopy	Confirmation of diagnosis
	X-ray	Concurrent infection, effusion and/or atelectasis
	MR lymphangiography	Localize leakage, visualize potential lymphatic abnormalities
Medical management		
	PDE-5 inhibitor	Pulmonary vasodilation, reduce lymphatic congestion
	Endothelin-1 inhibitor	Pulmonary vasodilation, reduce lymphatic congestion
	Carvedilol	Increase ventricular filling, lower end-diastolic pressure
	Diuretics	Reduce overhydration and lymphatic congestion
	Bronchodilator	Improve respiratory symptoms, mobilize casts
	Aerosolized hypertonic saline	Osmotic dilution and mobilization of mucus
	Mucolytics	Reduction of mucosal viscoelasticity, reduce and mobilize casts
	Unfractionated heparin	Prevent fibrin-crosslinking, anti-inflammatory, improve glycocalyx
	Fibrinolytics	Fibrinolysis, reduce cast size
	Corticosteroids	Anti-inflammatory
	(Leukotriene receptor inhibitors)	Anti-inflammatory
	(Macrolides)	Increased mucosal clearance, anti-inflammatory
Other management		
	Intensive chest physiotherapy	Mobilize casts
Surgical management		
	Bronchoscopy	Removal of casts
	Late fenestration	Reduce lymphatic congestion
	Surgical ligation of TD	Prevent thoracic lymphatic flow
	Lymphatic embolization	Block lymphatic leakage
	TD decompression (surgical or interventional)	Reduce lymphatic congestion, improve cardiac output
	Fontan take-down	Improve cardiac output, reduce lymphatic congestion
	Heart transplantation	Normalize circulation
Peripheral edema		
Diagnostic evaluation		
	Clinical examination	Confirm diagnosis
	(Lymphoscintigraphy)	Visualize lymphatic abnormalities
	(NIRF Lymphangiography)	Visualize lymphatic abnormalities
	(MRI Lymphangiography)	Visualize lymphatic abnormalities
	(Duplex ultrasound)	Examination for venous insufficiency
Management		
	Diuretics	Prevent overhydration and reduce edema
	Organ-specific treatment	Cardiac, hepatic and renal optimization
	Exercise	Reduce lymphedema
	Physiotherapy	Reduce lymphedema
	Compression bandaging	Reduce lymphedema
	Pneumatic compression therapy	Reduce lymphedema
